# Muscle performance and ankle joint mobility in long-term patients with diabetes

**DOI:** 10.1186/1471-2474-9-99

**Published:** 2008-07-04

**Authors:** Claudia Giacomozzi, Emanuela D'Ambrogi, Stefano Cesinaro, Velio Macellari, Luigi Uccioli

**Affiliations:** 1 Department of Technology and Health, Istituto Superiore di Sanità, Rome, Italy; 2 Internal Medicine Department, "Tor Vergata" University, Rome, Italy

## Abstract

**Background:**

Long-term patients with diabetes and peripheral neuropathy show altered foot biomechanics and abnormal foot loading. This study aimed at assessing muscle performance and ankle mobility in such patients under controlled conditions.

**Methods:**

Forty six long-term diabetes patients with (DN) and without (D) peripheral neuropathy, and 21 controls (C) were examined. Lower leg muscle performance and ankle mobility were assessed by means of a dedicated equipment, with the patient seated and the examined limb unloaded. 3D active ranges of motion and moments of force were recorded, the latter during maximal isometric contractions, with the foot blocked in different positions.

**Results:**

All patients showed reduced ankle mobility. In the sagittal and transversal planes reduction *vs *C was 11% and 20% for D, 20% and 21% for DN, respectively.

Dorsal-flexing moments were significantly reduced in all patients and foot positions, the highest reduction being 28% for D and 37% for DN. Reductions of plantar-flexing moments were in the range 12–15% for D (only with the foot blocked in neutral and in dorsal-flexed position), and in the range 10–24% for DN. In all patients, reductions in the frontal and transversal planes ranged 14–41%.

**Conclusion:**

The investigation revealed ankle functional impairments in patients with diabetes, with or without neuropathy, thus suggesting that other mechanisms besides neuropathy might contribute to alter foot-ankle biomechanics. Such impairments may then play a role in the development of abnormal gait and in the onset of plantar ulcers.

## Background

Diabetic peripheral neuropathy is the main cause of sensory and motor deficit of the feet: as a result, motor control of gait is compromised, nerve degeneration may cause muscle weakness and atrophy, and plantar ulcers may occur [1,2].

A further severe complication of diabetes, namely hyperglycaemia, should be also accounted for in the analysis of gait alterations. As a point of fact, hyperglycaemia alone promotes glycosylation of proteins, resulting in the accumulation of advanced glycosylation end-products in tissues. Damaging events will then ensue in almost every tissue and organ [3]. With respect to structures which are directly involved in gait, abnormal thickness of plantar fascia and Achilles tendon has been measured in long-term patients [4].

Several authors have dealt with the impairment of joint motion at the foot-ankle complex in the presence of diabetes. A general decreasing trend was observed in the range of motion, especially in flexion-extension movements [5,6]. Hypotheses were formulated about alterations in the structure of cartilages and capsules [7] which might interfere with joint mobility [5,8,9]. Studies were also conducted to investigate the role of muscular deficits in patients with diabetes [10-13]. Most *in vivo *studies analysed muscle performance under isokinetic conditions, both active [14-18] or passive [5,6]. In a recent study [13] on long-term diabetic patients under active isokinetic conditions, Andreassen highlighted a progressive muscle weakening, the rate of which was strictly related to the severity of neuropathy. Whereas in patients without neuropathy no significant change was found. Andersen [18] showed a significant muscle strength reduction in long-term patients with and without neuropathy; however, the data referring to non neuropathic patients were not separately reported in the paper. Since all the above measurements had been recorded during isokinetic exercises, other factors like inertia of the masses might have affected the measurements and masked some further findings.

Manual muscle testing (MMT) – a well known clinical method to assess muscle strength – has also been used to qualitatively assess muscle weakness in both ankle and knee of patients with diabetic neuropathy. However, its reliability is still doubtful; more specifically, it was found to significantly underestimate forces when compared with isokinetic dynamometry [19]. Thus, it does not seem an accurate method to investigate muscle performance better in long-term patients.

The present study aimed at accurately characterising the main functional alterations of the foot-ankle complex -namely joint mobility and muscle performance- in 46 long-term patients, 19 with and 27 without neuropathy, under well controlled conditions. An ankle dynamometer was used to assess: i) ankle mobility under active, unloaded conditions; ii) muscle performance of the main leg muscles during voluntary maximal isometric contractions.

From a clinical point of view, the study aimed at a deeper understanding of as many factors as possible which concur to alterations of gait parameters in the presence of long-term diabetes. Hopefully, the findings of the study may support the design of early rehabilitative paths aimed at preventing the onset of ulcerative processes.

## Methods

### Patient recruitment

Long-term patients with diabetes were recruited from the Outpatients Clinic of the University of Tor Vergata (Rome, Italy). Sixty one patients matched the inclusion criteria and were enrolled. Briefly, inclusion criteria were: age < 70 years; no history of peripheral vascular disease (ankle brachial pressure index > 0.85 and no symptoms of intermittent claudication); absence of neurological diseases but for those of diabetic aetiology; absence of muscular, skeletal or rheumatic disease; absence of any major or minor amputation; absence of Charcot neuro-arthropathy due to previous traumas [20]; diabetes duration > 7 years. The level of neuropathy was assessed by using the Neuropathy Disability Score (NDS) [21] and the Vibration Perception Threshold (VPT) [20]. For the purpose of this study, the presence of neuropathy was defined by NDS > 5 and VPT > 25 V [22]. Callouses, if any, were removed before measurements. Fifteen of the recruited patients had previously developed plantar ulcers that had healed at least three months before examination. Even though they were assessed and accounted for in other parts of the study, the analysis of their data is beyond the scope of the present investigation, and not discussed in the following. The remaining 46 long-term patients with diabetes were divided into group D (27 patients without neuropathy) and group DN (19 patients with neuropathy).

Twenty one healthy volunteers were screened and included in the study for comparison. They matched the above inclusion criteria but for specific, diabetes-related items. Their histories and the objective clinical examination excluded neuro-muscular or skeletal pathologies that might have influenced their gait.

Patients and healthy volunteers were recruited homogeneously as per sex, age, body mass index and occupation. Activity levels were only qualitatively assessed. Attention was paid to verify that both patients and healthy volunteers were routinely active and did not practice sports at a professional level.

All patients and healthy volunteers gave their informed written consent in accordance with the principles of Helsinki Declaration. The study was approved by the Ethics Committee of the University of Tor Vergata (Rome, Italy).

### The ankle measurement device

**Figure 1:**
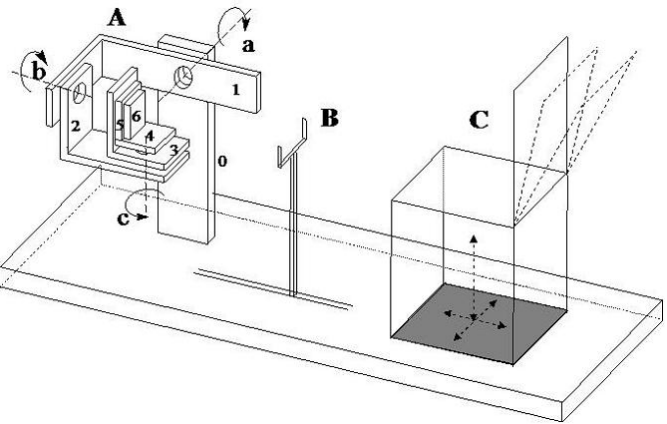
**Draft of the ankle measurement device**. **A**. Ankle measurement device (7 link, 6 DOF* mechanical chain); link 0 is solid with the patient's shank, link 6 with his/her foot. **B**. 2 DOF* metallic fork to maintain the knee in a fixed position. **C**. 3 DOF* adjustable seat, with bowable back. (a: flexion-extension axis; b: pronation-supination axis; c: internal-external rotation axis). * DOF = Degrees Of Freedom

**Figure 2:**
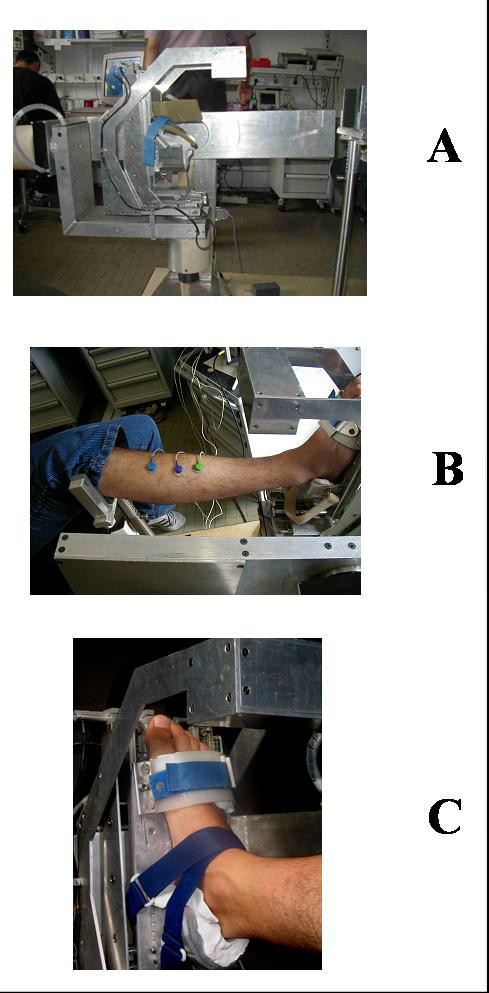
**The ankle measurement device**. **A**. Prototype of the ankle measurement device. **B**. Positioning phase of the right leg of a patient: the knee has already been fixed to the metallic fork; the forefoot has already been fixed to link 6 of the device; the rearfoot has been inserted in the *ad hoc *mould, and it only needs to be further fixed with Velcro stripes; the shank has already been aligned with link 0 of the device. **C**. Detail of a completed foot fixing.

The ankle device used to quantify joint mobility and muscular function had been designed and constructed at the authors' lab [23]. Briefly, it consists of a mechanical system which accurately measures linear and angular displacements of the foot with respect to the shank or, in case any rotation is blocked, moments of force at the foot-ankle complex expressed during isometric contractions. The device is accurately balanced, and the patient works under almost unloaded conditions. Figure 1 shows a sketch of the device. The prototype used in the present study is shown in Figure 2. Moments of force can be measured in any desired fixed position of the foot with respect to the shank. Measurement ranges are ± 250 Nm for flexion-extension, and ± 20 Nm for both inversion-eversion and internal-external rotation. The overall measuring chain, based on accurately calibrated strain gauge transducers, has high linearity (R2 = 0.993) and sensitivity (0.02–0.71 Volt/Nm).

Angular displacements can be measured in all three planes of the reference system by means of commercial, high-precision angular potentiometers (sensitivity 0.1 Volt/°). Measurement ranges are ± 100° for flexion-extension, and ± 50° for both inversion-eversion and internal-external rotation. All output signals are low-pass filtered at 10 Hz before being processed.

### Measurement protocol

During the measurement session the patient was asked to seat on an adjustable chair rigidly fixed to the wooden platform of the measurement device. His/her right shank was aligned to the first link of the device – previously rotated by +20° with respect to the horizontal plane – and the foot was fixed to the last link of the device: the forefoot was blocked with velcro stripes; the rearfoot was blocked by means of an aluminium block and an interposed polymethylsiloxane mould previously modelled on the patient's calcaneus. This blocking system guarantees for the free movement of the foot in the three planes. The foot was blocked at 90° with respect to the shank in the sagittal plane, and at 0° in the other two planes (frontal and transversal). The knee was tied to a fork-shaped support rigidly fixed to the base of the device.

The patients were first trained to perform and maintain maximal isometric contractions starting from resting condition. Then they were asked to perform a sequence of isometric maximal contractions – one for each measurement position – to produce moments around each axis, in both directions. Each contraction lasted for 10s and was followed by 90s of rest. The execution of the requested task was monitored by means of a surface EMG device. When abnormal muscle activity was observed before or during the contraction, the patient was asked to repeat the task after 90s of rest. Moments of force around the medio-lateral axis were further investigated by repeating the same exercise with the foot blocked at +15° and at +30° of plantar flexion, and at -15° of dorsal flexion. As regards active angular excursions, they were acquired sequentially in the directions of flexion-extension, internal-external rotation and inversion-eversion. Subjects were asked to perform slow cycles for a period of approximately 10s in each plane. They worked under almost unloaded conditions. No aid was supplied by the operator. A total of 6 or 7 cycles per plane were performed. The whole measurement sequence was repeated for the left leg.

### Data analysis

Data from right and left foot were processed separately for each patient. Maximum moments of force were normalised with respect to body weight and body height and expressed as %Nm. This normalisation was chosen to take into account not only the dependence of muscle length and moment arm on patient's height, but also their eventual dependence on his/her body mass.

Absolute values were used for angular excursions; the absolute maximum value in each direction of each plane (6 values per foot) was included in the study.

Once verified that they were normally distributed (chi-square test), basic parametric statistics (ANOVA with Bonferroni correction, p < 0.05) was applied to the above data and to the anthropometric and baseline clinical data of patients and volunteers.

## Results

The demographic data of the recruited population (Table 1) did not show significant inter-group differences as for age, metabolic control or diabetes duration, being the last two parameters only related to D and DN groups [22].

**Table 1:** Patients' baseline characteristics

		C	D	DN
Patients		21	27	19
Male/Female		13/8	19/8	10/9
Age (years)	mean (SD)	56.6 (11.8)	52.7 (12.7)	53.7 (10.4)
BMI (Kg/m^2^)	mean (SD)	25.0 (3.1)	25.3 (3.4)	27.0 (4.9)
Type 1/Type 2		-	8/19	9/10
Diab. Duration (years)	mean (SD)	-	15.1 (9.3)	19.4 (9.3)
HbA1c (%) °	mean (SD)	-	7.5 (1.5)	7.8 (1.8)
VPT toe (Volt)	mean (SD)	5.3 (2.1)	14.7 (5.7)	31.5 (7.6) *
NDS		0	3.8 [0–5]	6.95 [6–8]*
Retinopathy (a/b/p)		-	24/0/3	9/5/5
Nephropathy (a/m/M)		-	26/1/0	16/2/1

C: controls; D: diabetic patients without neuropathy; DN: diabetic patients with neuropathy

SD: standard deviation

* D *vs *DN; Student's t-test, p < 0.05

a/b/p = absent, background, proliferative;

a/m/M = absent/microalbuminuria/macroalbuminuria

° normal range: 4.3 – 5.9%.

Foot-ankle joint mobility showed an overall reduction in the sagittal and transversal planes (Table 2). Both groups of patients showed significant reductions (ANOVA with Bonferroni correction, p < 0.05) with respect to the control group, the only exception being plantar flexion in D patients (t = 2.222, critical t value = 2.355).

**Table 2:** Active joint mobility of the foot-ankle complex in all reference planes: columns 2–4 show mean values and standard deviations, expressed in degrees; columns 5–7 show t values obtained from the comparisons of C *vs *D, C *vs *DN and D *vs *DN (ANOVA with Bonferroni correction, p < 0.05, t critical value = 2.355).

	Mean value (SD)			t value of multiple comparisons		
	C	D	DN	C *vs *D	C *vs *DN	D *vs *DN

Dorsal flexion (p = 0.000)	34.4(8.3)	29.8 (6.4) *	27.3 (5.8) *	3.034	4.355	1.658
Plantar flexion (p = 0.000)	40.1 (7.0)	36.4 (5.1)	32.4 (7.1) *	2.222	4.209	2.291
Range (p = 0.000)	74.6 (13.6)	66.2 (11.3) *	59.6 (10.1) *	3.011	4.994	2.302
External rotation (p = 0.003)	41.8 (7.1)	34.6 (13.5) *	35.0 (10.0) *	3.222	2.779	0.192
Internal rotation (p = 0.000)	39.0 (7.4)	30.1 (14.6) *	28.9 (10.1) *	3.784	3.925	0.473
Range (p = 0.000)	80.8 (12.3)	64.7 (27.4) *	64.0 (18.3) *	3.710	3.553	0.159
Eversion (p = 0.018)	15.3 (5.1)	16.2 (6.8)	12.4 (4.9) §	0.688	2.044	2.830
Inversion (p = 0.707)	30.8 (6.9)	30.0 (10.4)	31.5 (8.1)	-	-	0.820
Range (p = 0.613)	46.2 (9.5)	46.2 (14.4)	43.9 (11.0)	-	-	0.901

C: controls; D: diabetic patients without neuropathy; DN: diabetic patients with neuropathy

SD: standard deviation

* C *vs *D or C *vs *DN (ANOVA with Bonferroni correction, p < 0.05 and t > 2.355)

§D *vs *DN (ANOVA with Bonferroni correction, p < 0.05 and t > 2.355)

A decreasing trend was observed with respect to muscle function (Tables 3 and 4). D and DN patients showed reductions of normalised moments of force in all planes, directions, and foot positions. For both groups the reductions were significant as for: i) dorsal flexing moments (sagittal plane) measured with the foot blocked at 0°, +15° and +30° (Table 3); ii) internal rotation in the transversal plane (external rotation significantly reduced for DN patients only); iii) eversion (frontal plane; Table 4). DN patients also showed statistical evidence of muscular impairment with respect to D patients as for plantar flexing moments exerted with the foot blocked at +15° and +30° (Table 3).

**Table 3:** Dorsal/plantar flexing moments at the ankle, normalised with respect to body weight * height.

	Mean value (SD)			t value of multiple comparisons		
	C	D	DN	C *vs *D	C *vs *DN	D *vs *DN

Plantar flexion at -15° (p = 0.205)	3.84 (1.27)	3.27 (1.99)	3.31 (1.53)	1.465	-	-
Plantar flexion at 0° (p = 0.328)	3.40 (1.36)	2.99 (1.50)	3.05 (1.26)	1.432	-	-
Plantar flexion at +15° (p = 0.009)	2.49 (1.14)	2.70 (1.40)	1.93 (0.84) §	0.863	2.115	3.075
Plantar flexion at +30° (p = 0.011)	1.40 (0.81)	1.50 (0.70)	1.06 (0.53) §	0.699	2.185	2.990
Dorsal flexion at -15° (p = 0.231)	2.40 (1.26)	2.17 (1.04)	1.96 (1.15)	-	1.718	-
Dorsal flexion at 0° (p = 0.002)	3.14 (1.34)	2.38 (1.31) *	2.21 (1.09) *	2.928	3.292	0.636
Dorsal flexion at +15° (p = 0.000)	3.38 (1.30)	2.45 (1.28) *	2.13 (1.09) *	3.658	4.518	1.223
Dorsal flexion at +30° (p = 0.000)	2.76 (0.85)	2.17 (0.99) *	1.88 (0.83) *	3.172	4.348	1.515

Mean values and standard deviations are expressed in %Nm. Moments are referred to four angular positions in the sagittal plane, with the foot blocked at: 15° of dorsal flexion (-15°); 0° (neutral position); 15° and 30° of plantar flexion (+15°; +30°). Columns 2–4 show mean values and standard deviations; columns 5–7 show t values obtained from the comparisons of C *vs *D, C *vs *DN and D *vs *DN (ANOVA with Bonferroni correction, p < 0.05, t critical value = 2.355).

C: controls; D: diabetic patients without neuropathy; DN: diabetic patients with neuropathy

SD: standard deviation

* C *vs *D or C *vs *DN (ANOVA with Bonferroni correction, p < 0.05 and t > 2.355)

§D *vs *DN (ANOVA with Bonferroni correction, p < 0.05 and t > 2.355)

**Table 4:** Internal/external torques and inversion/eversion moments at the ankle, normalised with respect to body weight*height.

	Mean value (SD)			t value of multiple comparisons		
	C	D	DN	C *vs *D	C *vs *DN	D *vs *DN

Internal rotation (p = 0.000)	1.12 (0.53)	0.83 (0.65) *	0.66 (0.38) *	2.575	3.753	1.467
External rotation (p = 0.003)	1.32 (0.50)	1.07 (0.69)	0.87 (0.47) *	2.105	3.482	1.636
Inversion (p = 0.278)	0.93 (0.36)	0.80 (0.48)	0.79 (0.49)	-	1.393	-
Eversion (p = 0.000)	0.73 (0.25)	0.55 (0.28) *	0.46 (0.25) *	3.332	4.593	1.619

Mean values and standard deviations are expressed in %Nm. Moments are referred to neutral position. Columns 2–4 show mean values and standard deviations; columns 5–7 show t values obtained from the comparisons of C vs D, C vs DN and D vs DN (ANOVA with Bonferroni correction, p < 0.05, t critical value = 2.355).

C: controls; D: diabetic patients without neuropathy; DN: diabetic patients with neuropathy

SD: standard deviation

* C *vs *D or C *vs *DN (ANOVA with Bonferroni correction, p < 0.05 and t > 2.355)

## Discussion

The experimental data of the present study showed a certain reduction of muscle performance at the foot-ankle complex in both groups of patients, DN and D, with and without neuropathy, respectively. In the sagittal plane the major impairment was found to be associated with the action of the dorsal flexors rather than the extensors of the ankle. The decrease ranged 10 to 28% in D patients and 18 to 37% in DN patients. In both groups the highest reduction was recorded with the foot blocked at +15° of plantar flexion.

Some changes in muscle structure and impairments in muscle function have been observed in presence of neuropathy and reported in the literature: i) muscle atrophy was measured at the level of the lower limb [10,11]; ii) muscle volume was found to be altered in long-term patients with diabetes and neuropathy, but preserved in the absence of neuropathy [10]; iii) a certain worsening in muscle performance was observed in patients with long-term diabetes [18] and peripheral neuropathy [13]. Most of the above studies were in a good agreement with the hereby reported findings as far as neuropathic patients are concerned. Some disagreement was found with respect to the hereby stated muscle weakness of long-term, non-neuropathic diabetic patients. The study by Andersen [18] did show a significant muscle strength reduction in the overall population of long-term patients, with and without neuropathy; however, the data referring to non neuropathic patients were not separately reported in the paper, therefore it was not possible to make direct comparisons.

The hereby calculated greater percentages of muscle strength reduction might be due to some differences in measurement instrumentation and procedures, the most important being the use of an isometric instead of an isokinetic test device. When performing isokinetic tests the patient has to work at a fixed angular velocity; this condition, however, is rigorously true for a limited time period of the exercise. At the beginning, in fact, a certain acceleration is allowed to the leg, until the isokinetic device reaches the selected value of velocity. Similarly, a certain deceleration is allowed in the final part of the exercise. The length of such "uncontrolled" phases depends on the established angular velocity: the higher the velocity, the longer the acceleration and deceleration phases. As a result, several variables, like inertial and gravitational effects, and the selected angular velocity may play a role in the overall recorded moment of force. In the present study the use of isometric and almost unloaded working conditions might have helped subjects do the task under better controlled conditions, thus highlighting even smaller differences in muscle performance, as it was for long-term, non neuropathic patients. Moreover, the assessment of muscle performance in the other two planes of the anatomical reference system showed that moments of force were significantly reduced also with respect to internal rotation and, most importantly, with respect to eversion, which confirms a significant muscular impairment of the ankle dorsal flexors. A reasonable explanation was the weaker performance of the dorsal flexors with respect to the extensors, more evident in D than in DN patients. As reported in [21], Achille's Tendon and Plantar Fascia showed abnormal thickness in D patients too. Thus, the hypothesis is that their joint action may mechanically result in a higher resistance to dorsal flexing moments.

As for the active joint mobility, the present study revealed its overall reduction in all planes and directions of movement, also evident in D patients with the only exception of the frontal plane. This finding is in agreement with the literature [5,6], and seems to confirm the hypothesis that the concurrent alterations of structure and coefficient of elasticity of cartilages and capsule may limit the active range of motion of long-term patients with diabetes.

To formulate a reasonable hypothesis about the functional impairment, the present study has demonstrated for patients without neuropathy, that moments of force may be conceived as the resultant of passive and active moments. A recent study [5] showed that a significant increase in the viscous component of passive ankle joint movement occurs in long-term diabetic patients, thus a certain percentage of the overall decrease in the moment of force may be ascribed to muscle atrophy induced by abnormal viscoelastic behaviour of passive motion. If present, this phenomenon is a direct consequence of tissue damages due to hyperglycaemia, and it is quite independent from neuropathy. Obviously, a major contribution to moment of force decrease is due to muscle atrophy directly related to nerve conduction degeneration, which is a direct consequence of neuropathy. Together with alterations of cartilages, ligaments and tendons [21], all these factors may explain the worsening of muscle performance, and may play a critical role in the concurrent – and strictly related – limitation of ankle active joint mobility, even in long-term patients without neuropathy.

The data reported in the present study were collected under controlled conditions altogether different from normal gait, i.e., with the patient seated and the foot unloaded. However, the hypothesis is hereby formulated that the resultant functional impairment and the adverse action of gravity may combine to seriously impair the biomechanics of gait. More specifically, they may worsen the management of both the landing and the propulsive phase of gait: in the former phase the foot is dorsi-flexed and the action of ankle flexors is required to slow down the leg and stabilise the foot before heel strike; in the latter phase the foot is plantar-flexed and the ankle extensors must deliver enough energy to correctly accelerate forward the centre of mass [24].

## Conclusion

Measurement equipments, protocols and methodologies were set up and specialised in the present study to assess the function of the diabetic foot-ankle complex under well controlled conditions, with the patient seated and the foot unloaded. The study focused on long-term patients with diabetes, with and without neuropathy.

As expected, and in agreement with the specialised literature, a certain reduction of 3D ranges of motion and moments of force was found in long-term patients with diabetes and peripheral neuropathy. Interestingly enough, significant functional impairments were found in the absence of neuropathy too.

In conclusion, the described foot-ankle functional assessment under strictly controlled conditions seems to be an accurate and effective means to highlight limitations in muscle performance and ankle joint mobility in long-term patients – even before the onset of neuropathy –. This suggests that other mechanisms besides neuropathy might contribute to alter foot-ankle biomechanics. Such mechanisms, and their role in the development of abnormal gait and in the onset of plantar ulcers, certainly deserve further investigation.

From a clinical point of view, the findings of the present study may help design *ad hoc *rehabilitative paths, in order to maintain an adequate level of gait performance in the presence of long-term diabetes, thus preventing excessive loading of foot tissues at risk of ulceration.

## Competing interests

The authors declare that they have no competing interests.

## Authors' contributions

CG led the design and implementation of the study, and performed the data analysis. ED participated in the design of the measurement protocol and in data collection and interpretation. SC participated in data collection and processing. VM and LU participated in the design of the study and in data interpretation. All authors actively participated in drafting the manuscript. They all read and approved the final manuscript.

## Pre-publication history

The pre-publication history for this paper can be accessed here:


